# Comparison of Phase Separation and Membrane Formation Behavior of Novel Amphiphilic Block Copolymers for Anti-Fouling Improvement of Ultrafiltration Membranes

**DOI:** 10.3390/membranes16050178

**Published:** 2026-05-19

**Authors:** Inga Frost, Oliver Gronwald, Mathias Ulbricht

**Affiliations:** 1 Lehrstuhl für Technische Chemie II, Universität Duisburg-Essen, Universitätsstr. 7, 45141 Essen, Germany; inga.frost@gmx.de; 2 Advanced Materials & Systems Research, Performance Polymer Blends & Membranes, RAP/ES, BASF SE, 67056 Ludwigshafen am Rhein, Germany; oliver.gronwald@basf.com

**Keywords:** poly(phenylene sulfone), poly(ether sulfone), fouling, ultrafiltration membranes, water treatment

## Abstract

The comparison of the ability of poly(phenylene sulfone) (PPSU), a recently introduced alternative membrane polymer, with established poly(ether sulfone) (PESU), both in combination with tailored amphiphilic block copolymer additives to improve ultrafiltration (UF) membrane separation and anti-fouling performance is the focus of this work. Different poly(alkylene oxide)-containing tri- and multiblock polymers with hydrophobic blocks analogous to the respective base polymer, PPSU or PESU, of varied length were used as additives in the casting solution. Membranes were subsequently prepared via film casting and a liquid non-solvent-induced phase separation (NIPS) process. The rheological properties and thermodynamic stability of the casting solutions were investigated. At the same mass concentration, PPSU-based casting solutions show overall higher viscosity that is also more sensitive to the presence of additives compared with PESU-based solutions. PPSU-based casting solutions also have lower tolerance to non-solvents. By adding certain block copolymers in ratios of up 10 wt.% relative to the base polymer, it is possible to increase the UF performance of the membranes of PPSU and PESU. An increase in the block length of the hydrophobic block of PESU leads to a reduction in pure water permeance (PWP), whereas for PPSU, PWP is increased by the addition of additives. Especially additives with shorter PESU or PPSU block length, i.e., with a larger fraction of poly(ethylene oxide) blocks in the casting solution, seem to act as additional pore-forming agents. The water contact angle can be decreased for both additive systems, indicating a more hydrophilic membrane surface. Finally, using flower soil extract as a model substance for surface water, interesting candidates of additives that enable fouling reduction with competitive UF performance were identified for PESU and PPSU membranes.

## 1. Introduction

Increasing industrialization worldwide, combined with climate change and a steadily rising global population, is leading to a shortage of fresh or drinking water. In the World Water Report 2021, the United Nations state over two billion people worldwide living in regions with water scarcity. Access to drinking water is therefore not available to about one-fifth of the world’s population [1,2,3].

Therefore, the use of alternative methods for treatment of fresh and waste water is of great interest. Already since the 1960s, membranes have offered an energy- and resource-saving alternative to conventional mechanical or thermal separation processes [4,5,6]. The main application areas of ultrafiltration are in drinking water production and wastewater treatment, in the food industry and in the biopharmaceutical industry [4,5].

UF membranes made of poly(ether sulfone) (PESU), poly(sulfone) (PSU) or poly(vinylidene fluoride) (PVDF) are the most widely established. In particular, PESU has the advantage of high mechanical and thermal stability and is easy to process into high-performance UF membranes [4,5]. In membrane applications, membrane fouling is a problem [6,7,8,9]. Consequently, membrane cleaning is necessary at regular intervals during operation to maintain consistently satisfactory performance. Mechanical backwashing but also chemical cleaning, for example, with hypochlorite solutions, are used as cleaning steps [10,11].

**Figure 1:**
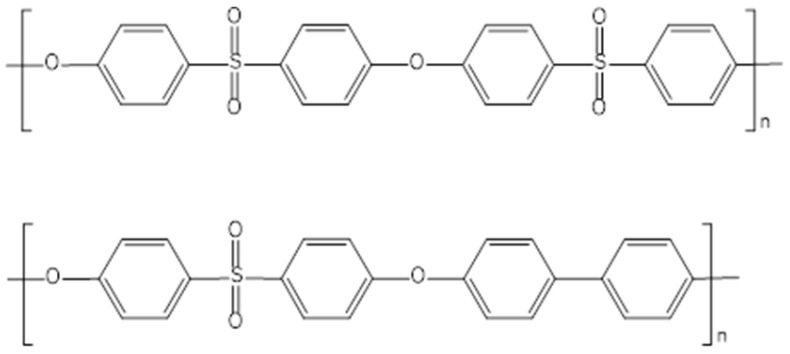
Structure of PESU and PPSU.

PESU membranes show degradation over time under the conditions of chemical cleaning, mainly by cleavage of the polymer main chain at the SO_2_ group [12,13]. It is therefore of interest to establish an alternative polymer for UF membranes. Poly(phenylene sulfone) (PPSU) contains only one SO_2_ group compared with the two SO_2_ groups in PESU in an otherwise identical repeating unit (Figure 1). This results in higher tolerance to oxidation agents and could thus positively influence the lifetime of the membrane modules under operation conditions [12,13]. However, PPSU, due to its higher hydrophobicity, is known to form denser membranes with lower UF performance under the same manufacturing conditions as for PESU [14]. Finding ways for facile improvement in UF performance of PPSU membranes may make them a potential material for water treatment.

Many studies on anti-fouling materials show that smooth, hydrophilic, and uncharged surfaces exhibit the lowest tendency to foul by natural organic matter (NOM). Several options exist to chemically modify membrane surfaces [15,16,17]. A simple and easily scalable method is the direct mixing of the membrane polymer with surface-modifying substances in the casting solution. It is known that amphiphilic block copolymers can accumulate on the membrane surface during membrane formation via the film casting of a polymer solution and subsequent liquid non-solvent-induced phase separation (NIPS) [18,19,20]. The hydrophilic block then leads to a reduction in fouling tendency, while the hydrophobic block effectively anchors the additive in the membrane matrix, thus ensuring the stability of the modification. At the same time, the additives can influence the formation of the membrane pore structure during the NIPS process. Gronwald et al. reported the successful use of PPSU-based amphiphilic block copolymers to improve not only the filtration performance but also to reduce the fouling tendency of PPSU UF membranes [21].

The aim of this work is to directly compare PPSU and PESU, in combination with different PPSU- or PESU-based amphiphilic block copolymers, with respect to membrane formation and the effects of the additives on membrane structure, UF separation performance and NOM fouling behavior. The novelty of this work is based on the systematic experimental exploration of the effects of additives from a library of tailored block copolymers with PESU or PPSU as anchor block and different hydrophilic polyethyleneoxide (PEO) blocks, to PESU or PPSU casting solutions on membrane formation, as well as the resulting membrane structure and properties. Additives with shorter PESU or PPSU blocks were found to more likely to act as pore-formers, and additives with longer PESU or PPSU blocks seemed to accumulate on the membrane surface, promoting hydrophilization and anti-fouling function of PEO-containing blocks. Overall, interesting candidates of additives that enable fouling reduction with competitive UF performance were identified for PESU and PPSU membranes.

## 2. Materials and Methods

### 2.1. Chemicals

Poly(phenylene sulfone) Ultrason P 3010, poly(ether sulfone) Ultrason E 3020P, polyvinylpyrrolidone Luvitec^®^ K90, 1,2-propanediol, and the block copolymer additives were obtained from BASF SE, Ludwigshafen, Germany, and used as received. Dextran 100 (number indicating average molecular weight in kg mol^−1^) was purchased from Serva GmbH, Heidelberg, Germany. Glycerol was purchased from Fisher Scientific GmbH, Schwerte, Germany. Potassium chloride, sodium bicarbonate and sodium hydroxide (1 M solution) were purchased from Bernd Kraft GmbH, Duisburg, Germany. Sodium carbonate was purchased from Carl Roth GmbH, Karlsruhe, Germany. N-Methyl-2-pyrrolidon was purchased from Merck Schuchardt OHG, Hohenbrunn, Germany. Sodium hypochlorite was purchased from abcr GmbH, Karlsruhe, Germany. Sodium thiosulfate was purchased from Acros Organics/Thermo Fisher Scientifics, Waltham, MA, USA. Ultrapure water was obtained from a Milli-Q^®^ water purification system from Merck KGaA, Darmstadt, Germany.

### 2.2. Membrane Base Polymers

Poly(ether sulfone) (PESU) and poly(phenylene sulfone) (PPSU) (see Figure 1) exhibit a high glass transition temperature (PES: 225 °C; PPSU: 220 °C) [21].

### 2.3. Block Copolymer Additives

Lutensol^®^ AT80 (BASF SE, Ludwigshafen, Germany), a monofunctional ethylene oxide fatty acid building block with an average molecular weight of 3780 g mol^−1^, was used for synthesis of triblock copolymer additives. The weight fraction of the poly(ethylene oxide) block in Lutensol^®^ AT80 is approximately 98 wt.%. For the synthesis of multiblock copolymer additives, a symmetrical poly(ethylene oxide)-block-poly(propylene oxide)-block-poly(ethylene oxide) (PEO-PPO-PEO) triblock copolymer (Pluronic^®^ F127, BASF SE, Ludwigshafen, Germany) was used. The total average molecular weight of pristine Pluronic^®^ F127 is 12,400 g mol^−1^. More detailed information can be found in the literature [21]. Table 1 gives an overview of the composition of the additives used, along with the PEO fraction according to ^1^H NMR spectroscopy.

### 2.4. Dope Solution Preparation

Table 2 gives an overview of dope solution composition.

For the reference dope solutions, 19 wt.% of the membrane base polymer was used. For membranes with low additive content, 0.4 wt.% was used, and for high additive content, 1.6 wt.% was used. Preliminary studies had revealed that in this range, effects of additives could be obtained without changes in the overall pore morphology of the membrane. The overall relatively small additive fractions are also interesting from the cost perspective, since water treatment applications with membranes must be implemented in a highly competitive market with cost as one of the main factors. PVP and PESU or PPSU were first weighed. It should be noted that PVP is sensitive to oxidation and was stored in an inert gas atmosphere prior to use. Subsequently, the additive was weighed into a 250 mL three-neck flask, and solvent NMP was added. Base polymer and PVP were then slowly added under stirring (KPG stirrer at low speed), and the flask was sealed with a stopper. Stirring at 60 °C was done until a homogeneous solution was obtained. In the final step, the non-solvent (glycerol or 1,2-propandiol) was added, and the solution was allowed to stir overnight at 60 °C.

Before use for the casting of membranes or further analysis, the solution was degassed in 100 mbar vacuum at 60 °C until no rising air bubbles were visible (min. 30 min).

With (Equation (1)) the theoretical hydrophilic component’s content through the additive in the dope solution was calculated.

(1) $${hydrophilic\;portion}_{calc}=\frac{portion\;of\;hydrophilic\;block\;in\;the\;dope\;solution}{portion\;ofPES\;or\;PPSU+portion\;of\;hydrophobic\;block}$$

### 2.5. Determination of Phase Diagram by Cloud Point Titration

To gain insights into sections of the ternary phase diagrams for PESU and PPSU, cloud point titrations were performed using water as non-solvent on polymer solutions of different concentrations. The following dilutions were used for PESU and PPSU in NMP:

1 wt.%; 5 wt.%; 10 wt.%; 15 wt.%; 20 wt.%; 25 wt.%.

From each sample, 5 × 10 g was weighed in vials, and increasing amounts of water were step-wise added (0.125–0.625 mL of ultrapure water). After each addition, samples were sealed airtight and shaken for at least 12 h. When the solution was clear again, another 0.125 mL of water was added, and the vial shaken again. When irreversible turbidity had been achieved in all samples, the amount of water added was evaluated, and the ternary phase diagram was generated from the data. Figure S1 (in Supplementary Information, SI) shows the typical appearance of polymer solutions with different non-solvent contents in the course of cloud point titrations.

For further characterization of actual casting solutions (cf. Table 2), cloud point titrations were carried out using the respective precipitation bath as non-solvent (cf. Section 2.7). In analogy to the procedure described above, defined amounts (0.125 mL) of the precipitation bath (glycerol/ultrapure water, 2:3) were added to the sample (5 g of casting solution), and the vial was shaken for 12 h. This was repeated until irreversible clouding of the casting solution occurred, and the cloud point could be noted.

### 2.6. Analysis of Rheological Behavior

The Physica MCR301 rheometer by Anton Paar GmbH, Ostfildern, Germany, was used to characterize the dynamic viscosity of the casting solutions. The geometry was conical with an angle of 1° and a sample holder diameter of 50 mm. The gap (50 μm) was adjusted and controlled via a magnetic field (TruGap by Anton Paar). For the measurement, 600 μL of the casting solution was applied to the sample holder, and the gap height was adjusted. Analysis was performed for each casting solution at 20 °C and 60 °C at 10 different shear rates, with a constant measuring time of 20 s per shear rate, as part of a logarithmic ramp with shear rates step-wise increasing from 0.1 s^−1^ to 800 s^−1^. Viscosity was also recorded at a shear rate of 40 s^−1^; this corresponds to the shear rate during the membrane casting process (see Section 2.7).

Furthermore, the viscosity at 1 s^−1^ was measured with a temperature ramp, from 10 to 60 ° C. The activation energy for molecular movement was calculated using the Arrhenius model (Equation (2)).

(2) $$\eta \left(T\right)=\eta_{\infty }\cdot exp\left(\frac{E_{A}}{RT}\right)$$

where *η_∞_* is a coefficient, *E_A_* is the activation energy, *R* is the universal gas constant and *T* is the absolute temperature.

### 2.7. Membrane Preparation

Membranes were prepared using film casting and NIPS. The Coatmaster 509 MC/1 from Erichsen GmbH, Hemer, Germany, located in a Plexiglass box with compressed air supply was used. This allowed the humidity (about 15% relative humidity at room temperature, RT) to be set and kept constant.

To ensure equal temperature, the glass plate, the doctor blade (300 μm gap height) and the casting solution were preheated to 60 °C, and this temperature was kept constant with the help of an oven. The casting solution was placed on the glass plate, cast into a film at a rate of 20 mm/s, and subsequently immersed in a coagulation bath consisting of glycerol/water (2:3 volumetric ratio) at room temperature. After 5 min, the membrane was transferred to a bath of DI water and stored for 2 h. Subsequently, round membrane samples 25 or 50 mm in diameter were punched out, and the samples were stored in ultrapure water until further processing.

To remove the remaining PVP, the membranes were treated under oxidative conditions in the next step [22]. First, a buffer at pH = 9.5 was prepared (6 vol% 0.2 M sodium carbonate, 20 vol% 0.2 M sodium hydrogen carbonate and 74 vol% ultrapure water). The membranes were shaken in a solution containing 2000 ppm of active chlorine, prepared from a sodium hypochlorite buffer (with 13–16% active chlorine), at 60 °C for 2 h. This was followed by washing with ultrapure water at 60 °C for an additional two hours. In the final step, the membranes were shaken in sodium thiosulfate solution (0.05 wt% in solution) at room temperature for 2 h. The membranes were then washed with ultrapure water. Storage was carried out in ultrapure water.

### 2.8. Filtration Performance Characterization

For determination of pure water permeance (PWP) and retention of dextran (average molecular weight: 100 kg mol^−1^) a setup consisting of a pressure tank, a water reservoir and three parallel dead-end Amicon stirred cells (model 8010, Merck KGaA, Germany) with a filter area of 4.1 cm^2^ was used. Using dextran with low fouling tendency is common for quantification of the size-selective properties of ultrafiltration membranes [19,23,24,25], and the specific molecular weight of dextran had been chosen because observed retention for all membranes was in the range of ~30 to ~90%, so that differences between the different membranes could be clearly detected.

The reservoir was filled with ultrapure water, and the membranes were installed in the filtration cells. First, the membranes were compacted for 30 min at a transmembrane pressure of 3 bar. Subsequently, the water flux was determined gravimetrically, three times for 5 min each, at a constant pressure of 0.5 bar. From this, PWP was calculated as follows (Equation (3)):

(3) $$PWP=\frac{V}{t\cdot A\cdot p}$$

where *PWP* = pure water permeance [L m^−2^ h^−1^ bar^−1^], *V* = filtered volume [L], *t* = filtration time [h], *A* = membrane area [m^2^], and *p* = transmembrane pressure [bar].

The cells were then emptied and filled with 10 mL of a 1 g/L solution of dextran in water. At a transmembrane pressure of 0.1 bar, 1.5 mL of filtrate was initially collected and discarded. Subsequently, 7 mL of permeate was collected for analysis. To determine the retention, the total organic carbon (*TOC*) content (Equation (4)) in feed and permeate was determined using the *TOC* system TOC-V CPN by Shimadzu GmbH, Duisburg, Germany. The retention was then calculated using (Equation (5)).

(4) TOC=TC−TIC

where *TC* = total carbon content [mg L^−1^], *TIC* = total inorganically bound carbon [mg L^−1^], and *TOC* = total organically bound carbon [mg L^−1^].

(5) $$R=\left(1-\frac{{TOC}_{P}}{{TOC}_{F}}\right)\cdot 100$$

where *R* = retention [%], *TOC_F_* = organic carbon concentration in feed [mg L^−1^] and *TOC_P_* = organic carbon concentration in permeate [mg L^−1^].

### 2.9. Scanning Electron Microscopy (SEM) Analysis

First, solvent exchange was performed for drying the membranes. This involved a step-wise exchange from water to ethanol, followed by the air drying of the membranes. Thereafter, dry membrane samples were frozen in liquid nitrogen and then broken. The surfaces to be examined were sputtered with AuPd for 30 s using the Sputter Coater Cressington MTM 10, Micro to Nano BV, Harleen, The Netherlands, (sputter target: Au80Pd20) at approximately 4–5 nm thickness and then analyzed with a Quanta 400 FEG ESEM instrument by Thermo Fisher Scientific, Waltham, MA, USA.

### 2.10. Contact Angle Determination

Sessile-drop contact angle experiments were performed on the dry membranes (cf. Section 2.9) to characterize the surface hydrophilicity. Measurements were performed using the Contact Angle System OCA 15 Plus by DataPhysics GmbH, Filderstadt, Germany, at a temperature of 22 °C and using ultrapure water. A droplet with a volume of 5 µL was applied, and the fitting of the contour was performed according to Young–Laplace. At least two samples were examined for each membrane type. The contact angles specified in this work are the average values of at least 5 measurements per sample.

### 2.11. Zeta Potential Analysis

The zeta potential of the membrane surface was determined using a SurPass Electrokinetic Analyzer System by Anton Paar GmbH, Ostfildern, Germany. For this purpose, two round membrane samples with a diameter of 14 mm were punched out and attached to the two sample holders with double-sided adhesive tape. In the measuring cell, a gap of 100 µm between the samples was set. The proper alignment of the sample holders relative to each other was confirmed via the “flow check” (linearity of flow as a function of pump pressure). A 0.001 M KCl solution, which was adjusted to a pH of 3 with 1 M hydrochloric acid, served as the electrolyte. After step-wise pH titration with 1 M NaOH up to a pH value of 10, the streaming potential at different specific pH values was measured, from which the zeta-potential was determined according to the Helmholtz–Smoluchowski model.

### 2.12. Adsorptive Fouling Experiments

**Figure 2:**
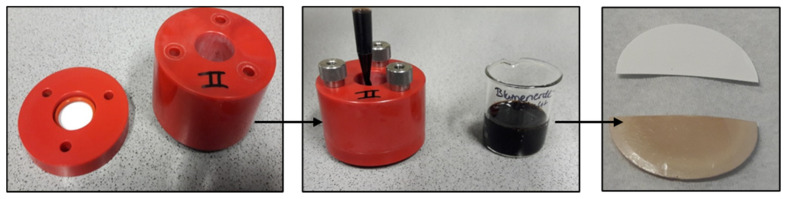
Visualization of adsorptive fouling experiments; the left and middle image show the membrane sample holder and liquid reservoir, the right image shows pieces of a fouled and an unfouled reference membrane.

To characterize the fouling behavior of the membranes, a system of potting soil extract was adapted to the requirements of this work. Due to its chemical composition, potting soil extract resembles the composition of humic substances in surface water and was therefore selected. The extraction procedure and resulting potting soil extract composition had been described by Kouchaki Shalmani et al. [23]; it was followed during this study. The purpose of the fouling test was to quantify the effect of adsorption of foulant on the membrane surface, i.e., without flux through the membrane, on permeance [24,25]. A series of tests were first carried out to establish standard conditions, which were suited for the specific membranes. A relative flux reduction (RFR) due to a fouling of 30% was set as the target for the reference membrane without additive. To determine the fouling behavior, the membrane was first compacted, and PWP was determined (cf. Equation (3)). Subsequently, the membrane was installed in a special cell with the active side facing upwards; see Figure 2.

Subsequently, 3 mL of a 1:1 mixture of potting soil extract with DI water (pH = 7) was added to the membrane and shaken for 24 h. Thereafter, *PWP* was determined again, and the *RFR* value was calculated (Equation (6)).

(6) $$RFR=(1-\frac{{PWP}_{after}}{PWP_{before}})\cdot 100$$

Above, *PWP_after_* = *PWP* after fouling [L m^−2^ h^−1^ bar^−1^] and *PWP_before_* = *PWP* before fouling [L m^−2^ h^−1^ bar^−1^].

## 3. Results and Discussion

### 3.1. Casting Solutions

#### 3.1.1. Solubility and Miscibility Effects

**Figure 3:**
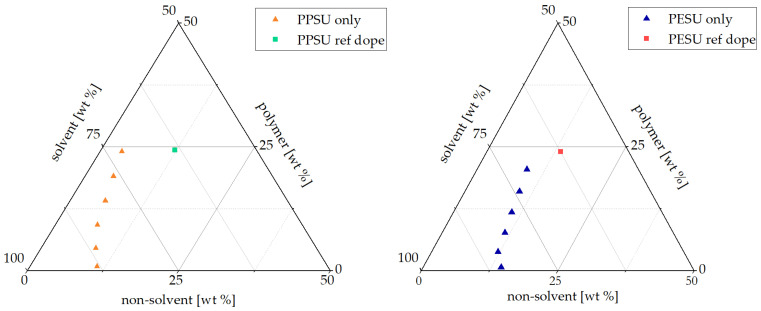
Ternary phase diagrams for PPSU (**left**) and PES (**right**) at 20 °C. Additionally, each reference dope solution exposed to the non-solvent with the composition of the coagulation bath are marked in the diagram.

For the fabrication of the reference membranes, a relatively complicated composition of the casting solution (cf. Table 2) and a special coagulation bath (glycerol/water, 2:3; cf. Section 2.7) were used. This was based on results of separate previous studies (for PESU, see [26]; for PPSU, see [21]), with the aim to obtain membranes with a sponge-like cross-section morphology. In order to quantify the difference between the two membrane polymers in terms of their interactions with the main solvent and non-solvent, Figure 3 presents parts of the ternary phase diagram for PPSU and PES in NMP and water (unlike the casting solution, these systems do not contain PVP and glycerol or 1,2-propandiol). A PESU solution with a polymer fraction similar to the casting solution tolerated up to 10 wt.% water. With the increase in polymer concentration, a decrease in non-solvent tolerance was observed. Compared with PESU, the binodal for the analogous PPSU system was clearly shifted to lower non-solvent contents; with higher polymer concentrations, tolerance to the non-solvent also decreased.

The cloud points of casting solutions for the reference membranes, without block copolymer additive, when combined with a non-solvent having the composition of the coagulation bath were determined to obtain information on their thermodynamic stability. For the casting solutions, PVP on the one hand and glycerol for PESU and 1,2-propanediol for PPSU on the other hand were included (see Section 2.4). For the representation in the diagram, the components are assigned as follows: PESU or PPSU and PVP are accounted for in the polymer portion, and glycerol or 1,2-propanediol and water are considered non-solvents. The difference in the thus represented cloud point with respect to the binodal composition for the PPSU/PESU-NMP–water systems was much bigger for the PPSU system compared with the PESU system.

The strength of a solvent can be described via the Hansen solubility parameters (HSPs) [16,27,28,29]. The smaller the differences between the individual contributions to solubility and their sum parameter (δ) for two components are, the better they mix. Table 3 shows the Hansen parameters for all components used or relevant in this study.

Between the two membrane polymers, PESU is generally more hydrophobic than PPSU, as indicated by the smaller HSP difference (Δδ) with respect to water for PPSU compared with PESU. This suggests that PPSU has stronger interactions with water because of its higher polarity (δ_p_). The aromatic structure of PPSU leads to higher polarity due to direct bonding between two aromatic rings, unlike for PESU, where SO_2_ groups contribute more to hydrogen bonding (δ_h_) [32]. Nevertheless, the overall difference between PESU and PPSU relative to water is small.

NMP, as indicated by its relatively small Δδ values for both PESU and PPSU, is a good solvent for both polymers but slightly better for PESU. This is likely due to the matching polarity and hydrogen bonding abilities of NMP with respect to PESU. PVP, while showing higher Δδ values, still exhibits compatibility with both PESU and PPSU.

PEO, on the other hand, shows good compatibility with both PPSU and PESU, as indicated by the relatively low Δδ values. This suggests that PEO can interact effectively with both polymers, likely due to a balance of polar and dispersive interactions, making it a suitable additive in membrane applications.

Experimentally it was observed that the tolerance of PPSU solutions in NMP to the addition of water was significantly lower compared with that of the analogous PESU solutions (Figure 3). Considering the Hansen parameters, this was not because PPSU is the more hydrophobic polymer (according to Δδ, PESU is slightly more hydrophobic) but because NMP is a somewhat poorer solvent for PPSU than for PESU.

Furthermore, the interactions between PESU and PVP are stronger than those for PPSU and PVP, mainly due to the high contribution of polar interactions for PPSU. The pronounced miscibility of PESU or PPSU with PVP is the reason for considering both to be solid polymer components when showing the cloud points of the reference casting solutions in the phase diagrams (cf. Figure 3).

A polymer-specific non-solvent was used for both casting solutions, and the non-solvent strength of the coagulation bath was also modulated [33,34]. Compared with glycerol, 1,2-propanediol has only two OH groups and is thus less hydrophilic. The non-solvent strength can be quantified by the HSPs and has the following order: water > glycerol > 1,2-propanediol.

In an earlier study [26], it was found that the addition of the non-solvent glycerol to a PESU-containing casting solution caused the system to move closer to the binodal; i.e., less non-solvent was needed to cause phase separation. It was also shown that the addition of glycerol promoted the formation of a sponge-like pore morphology of the membrane [26]. The much bigger difference in the cloud point with respect to the binodal composition for the PPSU system compared with the PESU system (cf. Figure 3) can easily be explained by the fact that in the PPSU system, a weaker non-solvent (1,2 propanediol) was used compared with that (glycerol) used for the PESU system.

The tolerance to the non-solvent decreased with the increase in polymer concentration in a similar manner for PESU and PPSU. The polymer concentration is crucial to the phase separation process leading to the porous membrane. Strathmann and Kock [35] showed that a higher polymer concentration tended to lead to the formation of a sponge-like pore morphology. They explained this with the higher polymer concentration at the interface between cast polymer solution and coagulation bath, and the resulting slower precipitation of the membrane underneath this interface. The addition of glycerol to the aqueous precipitation bath lowered the non-solvent strength, leading to slower coagulation; this also favored the formation of the desired sponge-like pore morphology.

#### 3.1.2. Rheology

**Figure 4:**
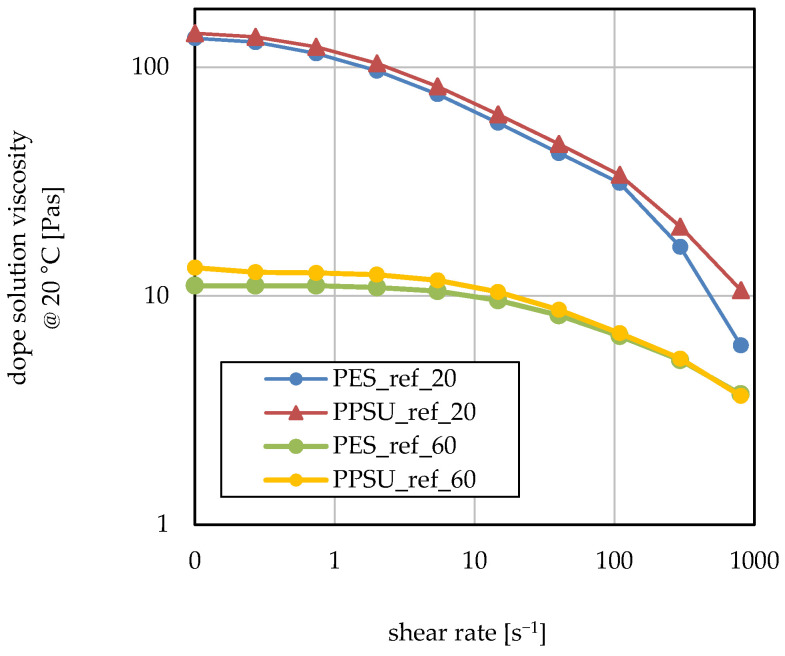
Casting solution viscosity as a function of shear rate and temperature (20 °C vs. 60 °C) for PESU and PPSU reference dope solutions.

The behavior of all casting solutions was investigated using a shear rate ramp in a range between 0 s^−1^ and 800 s^−1^ at 20 °C and 60 °C. Figure 4 shows the viscosity versus the shear rate for the PESU reference and PPSU reference casting solutions (i.e., with PVP but without block copolymer additive). Both solutions exhibited very pronounced shear thinning behavior. Overall higher viscosity was observed for PPSU than for PESU. At higher shear rates, the viscosity values converged. At 60 °C, the differences were smaller, but the trend remained the same.

Because of the high polymer content in the casting solutions, their rheological behavior is expected to be that of systems in the “concentrated regime”. The uncoiling of the polymer chains with the increase in shear rate leads to a very pronounced decrease in viscosity [36]; this is in line with the observed obvious shear thinning behavior (cf. Figure 4).

The average molecular weight (Mw) of PESU is 52 kDa, and that of PPSU is 48 kDa. Hence, at the same concentration, PPSU solutions would be expected to show slightly lower viscosity. According to the slightly lower solvent quality of NMP for PPSU compared with PESU, lower viscosity would also be expected; however, the additional effect of the different non-solvents likely also plays a role. On the other hand, one possible reason for the observed higher casting solution viscosity for PPSU could be the stiffer polymer backbone. The additional SO_2_ group in the PESU backbone leads to higher chain flexibility. The lack of one SO_2_ group in the PPSU repeating unit also enables more effective π-π-stacking interactions between chain segments; when this occurs between different macromolecules, it results in higher viscosity [14].

One measure used to assess the effects of inter- and intramolecular interactions is the activation energy for molecular motion, which can be obtained from an Arrhenius analysis of the viscosity as a function of temperature [14]. Figure S2 (see SI) shows the plot of all data for the PESU and PPSU reference casting solutions (with PVP but without amphiphilic additive). Activation energies were determined to be 37.9 kJ/mol for PESU and 40.9 kJ/mol for PPSU. These results confirm the above-discussed differences between PPSU and PESU as a reason for the higher viscosity [14,37,38,39].

**Figure 5:**
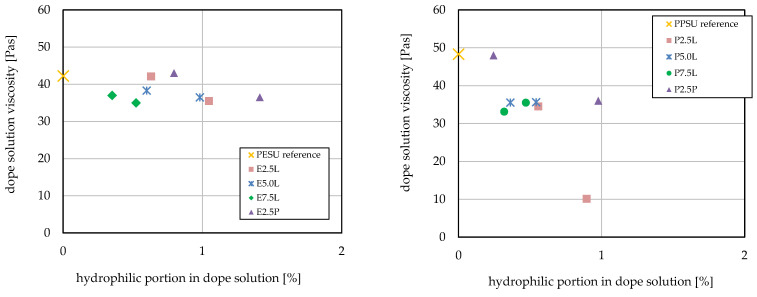
Comparison of viscosity at 20 °C and a shear rate of 40 s^−1^ for dope solutions with different amphiphilic additives for PESU (**left**) and PPSU (**right**).

All other casting solutions with block copolymer additive also exhibited very pronounced shear thinning behavior and the same strong dependency of viscosity on temperature. Viscosity at a constant shear rate of 40 s^−1^ was evaluated to reveal differences between the different casting solutions more clearly. The results are presented in Figure 5.

For the series of Lutensol-based additives, the increasing PESO or PPSU block length caused a reduced hydrophilic fraction of the additive in the casting solution; this hydrophilic fraction is in all cases composed of poly(ethylene oxide) (see Section 2.3). For PESU and the 2.5 k PESU-Lutensol additive in a low proportion in the casting solution, viscosity comparable to the reference solution was observed. This was also true for the Pluronic-based additive in small proportion. For all other combinations, the additive reduced the casting solution viscosity. For 7.5 k PESU-Lutensol and the Pluronic-based additive at high content, the lowest viscosity values were observed; the values decreased from 42 Pas for the reference solution to 35 Pas for such solutions with additive. For PPSU, higher viscosity was found for the reference casting solution compared with PESU. The addition of Lutensol-based additives lowered the viscosity for all combinations compared with the reference. The 2.5 k PPSU-Lutensol additive in high proportion resulted in a very low viscosity of 10 Pas compared with the 48 Pas of the reference solution. The Pluronic-based additive in low proportion caused no change; with a higher proportion of additive (1.6 wt%), the viscosity decreased to 36 Pas.

For the PESU systems, relatively small influences of the amphiphilic additive were observed. These could be due to the fact that the hydrophilic fraction, i.e., poly(ethylene oxide), as a weak non-solvent, destabilizes the solution state of the membrane polymer and leads to lower viscosity. According to the HSPs, the difference between PESU and PEO is relatively small (see Table 3); i.e., the destabilizing effect is relatively minor.

Compared with PESU, dissolved PPSU is more susceptible to perturbation of the interactions by non-solvents (see Section 3.1.1). The hydrophilic fraction, poly(ethylene oxide), as indicated by the difference in HSPs (cf. Table 3), is also less compatible with PPSU compared with PESU. Therefore, the overall effect, a stronger impact leaning toward lower viscosity, can be explained. In that frame, it can also be understood that differences in additive structure also have a bigger influence. With a short PPSU block and at higher additive fractions, the viscosity was markedly lowered. The longer hydrophobic segments in the 5.0 k and 7.5 k PESU-based additives provided better tolerance to the destabilizing effect of the Lutensol block, thus leading to a smaller effect on viscosity. Compared with the Lutensol-based additives, the Pluronic-based block copolymer did not reduce viscosity as much at comparable hydrophilic content. This can be attributed to its more pronounced amphiphilic character; hence it acts as a weaker non-solvent.

#### 3.1.3. Conclusion: State of the Casting Solutions

**Figure 6:**
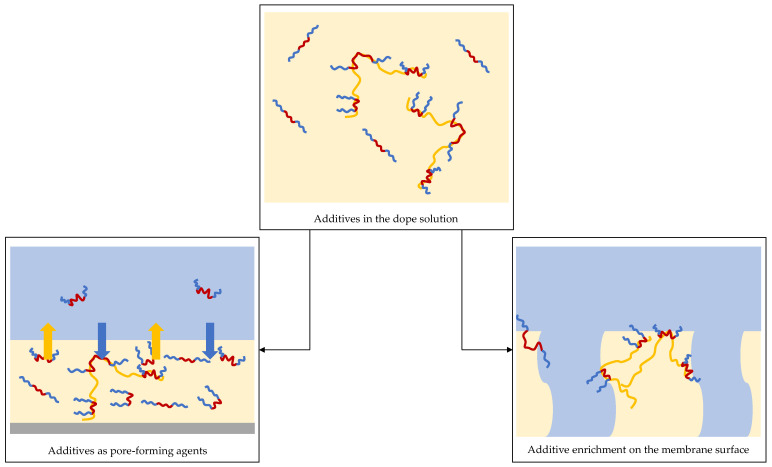
Two possibilities for the amphiphilic block copolymer additive function during membrane formation via NIPS (hydrophilic and hydrophobic segments in block copolymer in blue and red color, respectively; membrane polymer in yellow color; blue arrow indicates flow of water into the cast film, yellow arrow indicates flow of solvent out of the cast film).

The addition of hydrophilic poly(ethylene oxide) as part of the amphiphilic block copolymers reduced the casting solution viscosity. This was observed to be stronger for PPSU, which is more sensitive to the addition of non-solvents (cf. Section 3.1.1). The use of additives with longer PESU or PPSU segments (at the same mass fraction) mitigated the effect of destabilizing the solution. Based on the results, two different modes of action of the block copolymer additives on the membrane pore structure and surface properties are proposed (see Figure 6):

1. Function as additional pore-formers, thus changing the membrane pore structure;
2. Accumulation at the interface, thus changing the membrane surface properties.

For larger PESU blocks, the accumulation at the membrane surface may be more pronounced, potentially slowing down phase separation. This could result in a denser membrane barrier layer, leading to reduced permeance. For the Pluronic-based additive, this model requires extension, since the additive itself is an amphiphilic triblock copolymer. The hydrophobic segments of Pluronic may interact with PESU, potentially amplifying the aforementioned effect [40,41].

For PPSU as the membrane polymer, the lower stability of the casting solutions toward the non-solvent suggests a more open membrane barrier pore structure, even at low additive fractions in the casting solution. This difference aligns with the stronger response of PPSU to hydrophilic fractions in the additive and can be attributed to its higher susceptibility to destabilizing effects during phase separation.

### 3.2. Membrane Structure

**Figure 7:**
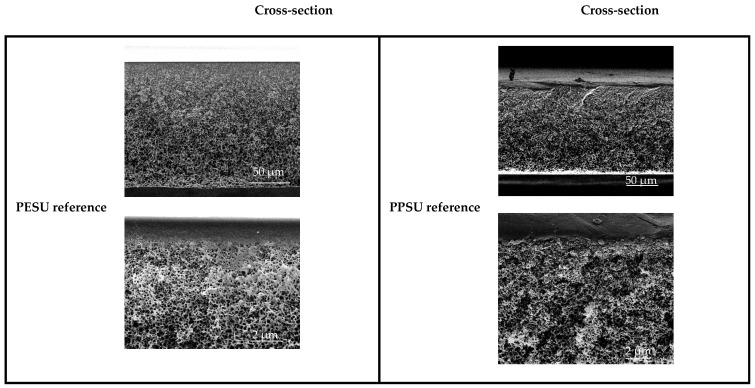
SEM cross-section for PESU and PPSU reference membrane.

Figure 7 provides an overview of the SEM cross-sections of the PESU and PPSU reference membranes. An anisotropic sponge-like structure was found for both membranes. The membranes have no finger pores or other undesired artefacts. The barrier layer is denser than the underlying support structure, as can be seen in the close-up of the active side surface. In additional top-side SEM evaluations, the active surface was found to be very smooth and regular. Based on the acquired SEM data, a quantitative analysis of surface pore structure (pore size and porosity) did not yield results that enable a meaningful comparison between the different membranes. Exemplary SEM images of the top surfaces are presented in Figure S3 (SI). The use of the different additives did not result in a significant change in the cross-section structures. Representative images for the respective reference membranes without additive are shown in Figure 7. Overall, the results are in line with other work [21,26] and confirm that the chosen NIPS conditions are suited to obtain UF membranes with the desired anisotropic cross-section morphology.

If the additives accumulate on the membrane surface (cf. Figure 6), the presence of PEO chains is expected to increase the surface hydrophilicity. However, for the additives employed in this study (amphiphilic block copolymers combined with PVP), the effect of post-treatment on surface structure and properties (see Section 2.7) must also be considered. Gronwald et al. [21] analyzed the inside barrier surface of hollow-fiber membranes fabricated from analogous PPSU-based casting solutions; they could confirm that poly(alkylene oxide) content at the surface determined by XPS was proportional to the poly(alkylene oxide) fraction in the casting solution due to addition of the block copolymer. XPS analyses of the top surface of the membranes in the present study yielded results that were below the detection limit of the method.

**Figure 8:**
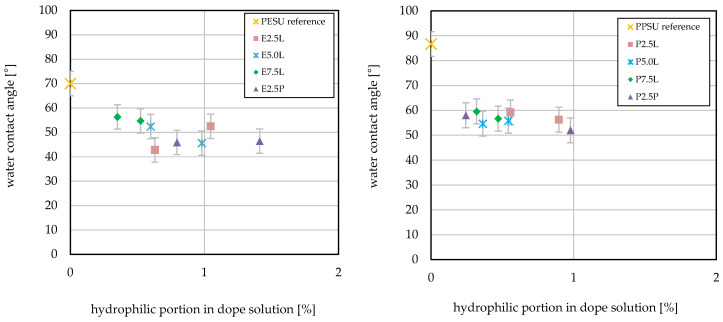
Water contact angle plotted versus the hydrophilic fraction of the additive in the casting solution for PESU (**left**) and PPSU ((**right**); according to two-sided Student’s *t*-tests, all modified membranes exhibit significantly lower contact angles relative to the unmodified reference membrane (*p* < 0.05); according to one-way ANOVA analyses, for most of the pair-wise comparisons between different membranes, the differences are not statistically significant).

To determine the surface hydrophilicity of the membranes, water contact angle measurements were carried out in sessile-drop mode. Figure 8 shows the data as a function of the hydrophilic fraction of the additive in the casting solution.

The PESU reference membrane showed a contact angle of 70°. All membranes prepared with block copolymer additive exhibited significantly lower contact angles. However, the additive structure had an influence: membranes containing the 2.5 k PESU-Lutensol additive had smaller contact angles compared with those with longer PESU segments (5.0 k and 7.5 k), and smaller contact angles were found for the 2.5 k PESU-Lutensol-containing membranes compared with the ones containing additive with longer PESU segments (5.0 k and 7.5 k). Specifically, as the PESU block length increased, the contact angle increased, though the difference between 5.0 k and 7.5 k PESU was minor compared with the 2.5 k variants. The lowest value of 43° was obtained for E2.5L at low fraction in the casting solution. Beyond this slight effect of block length, no clear dependence of the contact angle on the kind of additive or the additive content was recorded. For the Pluronic-based additive in combination with PESU, a reduced contact angle of around 42° independent of the proportion in the casting solution was found. For PPSU the water contact angle decreased from 58° to 52° with P2.5P with the increase in the amount of additive in the casting solution.

Compared with PESU, the contact angle for the PPSU reference membrane was higher, with a value of 87°. For all membranes prepared with additive, a lower contact angle was found. In contrast to PESU, no difference was found for the additives with different block length of PPSU. For all three PPSU/Lutensol additives, the average contact angle was 55°. For the Pluronic-containing membranes, no clear correlation between additive content and contact angle was observed.

For PESU, all additives lowered the water contact angle (cf. Figure 8). Increasing the barrier pore size and/or porosity can also lead to a decrease in the contact angle. Because such changes can indeed be discussed based on ultrafiltration data (see Section 3.3), the increase in barrier pore size appears to contribute to the lower contact angles. Therefore, the hydrophilic components appear to modify the surface, but no clear relationship between the kind of hydrophilic block and its fraction in the casting solution and the water contact angle could be identified.

The PPSU reference membrane exhibited a significantly higher water contact angle compared with the PESU membrane, indicating a more hydrophobic surface. This behavior can be attributed to the smaller fraction of SO_2_ groups in the PPSU polymer chain. Although this observation initially seems contradictory with respect to the HSP values (Table 3), it can be rationalized by the key role of SO_2_ groups in hydrogen bonding, particularly with water. Consequently, PPSU is inherently more hydrophobic. Due to this higher baseline contact angle, the reductions observed for PPSU membranes with additives were more pronounced than for PESU membranes. However, the contact angle values across all PPSU membranes were similar, showing no clear correlation among block length, additive fraction, and contact angle.

Figure S4 (see SI) shows the zeta potential as a function of the pH value for the PESU reference and the PPSU reference membranes, data that are typical for all membranes. No isoelectric point was found; in the whole pH range studied (pH = 2 to 10), a negative zeta potential was observed, with values of −127 mV at pH = 3 and −217 mV at pH = 10 for PPSU, and −90 mV at pH = 3 and −193 mV at pH = 10 for PESU.

For porous membranes, it is known that the tangential flow across the surface may also lead to some tangential flow through the pores of the membrane. As a result, the absolute value of the measured zeta potential is often found to be higher than the real value [24,42,43]. PESU UF membranes typically exhibit a negative zeta potential, which decreases with the increase in pH values. Since PESU is an intrinsically uncharged polymer, this is explained by preferential non-specific adsorption of anions on the surface. The concentration of OH- increases with the increase in pH, so that a more negative zeta potential is obtained [24,44,45]. Very high absolute values of zeta potential were found for the membranes in this study. Post-treatment of the membranes by hypochlorite oxidatively degrades the PVP that is still present in the membrane after NIPS and washing. While this process and the effect onto PESU UF membrane structure and separation performance has been investigated in detail [46], to our knowledge, no analogous study has been performed on PEO-modified PESU membranes. However, it is known that surface-grafted PEO is also not fully stable under such oxidative conditions [47,48]. The degradation products can contain charged groups such as carboxylate. In case the degradation products of PVP are not fully washed out, this could explain the very high absolute values of the zeta potentials. The actual surface structure would then be composed of the polysulfone matrix and entangled neutral poly(ethylene oxide), as well as charged macromolecular segments, leading to the observed hydrophilic properties.

Considering the scope of this study, a dedicated analysis of the mechanical properties of the membranes was not performed. However, no indication of mechanical instability of the flat-sheet membranes was observed during laboratory handling and testing, indicating a good synergy between the intrinsic properties of the membrane polymers PESU and PPSU and the accomplished homogenous sponge-like pore morphology.

### 3.3. Ultrafiltration Performance

**Figure 9:**
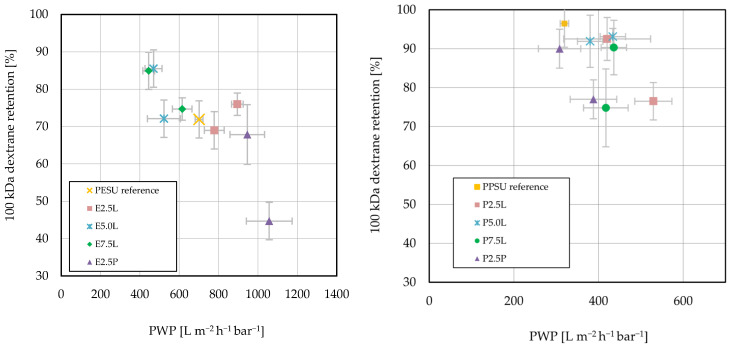
Comparison of ultrafiltration performance of membranes with different additives for PESU (**left**) and PPSU (**right**).

Figure 9 shows the comparison of the ultrafiltration performance for PESU- and PPSU-based membranes prepared with the different additives.

For PESU, the addition of the 2.5 k Lutensol additive provided an increase in PWP, while dextran retention remained within the range of the reference membrane. With larger additive content, a maximum PWP of 895 L m^−2^ h^−1^ bar^−1^ was achieved. Adding the additives with longer PES blocks in combination with Lutensol led to a decrease in PWP. With larger additive content in the casting solution, dextran retention was increased to 85%. The addition of the Pluronic-based additive led to a deterioration in filtration performance at both concentrations, with the highest PWP being obtained at only 42% dextran retention for the high additive content.

For PPSU, a lower PWP of the reference membrane compared with PESU at higher dextran retention (>90%) was observed. For the 2.5 k and 7.5 k Lutensol PPSU additive at high content in the casting solution, dextran retention was less than 80% with a simultaneous increase in PWP. The addition of the Pluronic-based additive led, similarly to PESU, to a reduction in dextran retention. In contrast to PES, however, the PWP was not significantly increased compared with the reference.

The ultrafiltration performance of a membrane primarily depends on barrier pore size and distribution and the thickness of the separation layer. Surface hydrophilicity may have an additional effect. The barrier structure is determined during the NIPS process, and thermodynamic boundary conditions and kinetics of phase separation in this study are influenced by the variations in the casting solution composition (cf. Section 3.1.1 and Section 3.1.2). Tests of all membrane types via gas flow/pore dewetting permporometry were performed, but a “bubble point”, corresponding to a barrier pore size of >~20 nm, was not detected. Hence, the contribution of defects in the barrier layer to enhanced permeability is very unlikely.

According to the SEM analyses (cf. Section 3.2), all membranes showed sponge-like pore morphology. Therefore, the additives do not seem to destabilize the casting solutions beyond a critical point at which instantaneous demixing occurs. The interactions of PESU or PPSU blocks of the block copolymers with the respective membrane polymer seem to overcompensate for influence of the hydrophilic blocks.

A well-known problem for membranes is the trade-off relationship between water permeance and solute retention [49]. With a view to use UF membranes in typical applications, for example, water treatment, it is therefore desirable when a modification leads to an increase in permeance at the same retention. The classification of the membranes according to the data shown in Figure 9 is discussed below.

For PESU and Lutensol additives with longer PESU blocks (E5.0L and E7.5L), retention was maintained or improved, but at lower PWP, which is undesirable. The membranes obtained with the Pluronic-containing additive (E2.5P) had higher permeance at reduced retention, i.e., revealing the same trade-off as almost all membranes. Only Lutensol-containing E2.5L stood out by leading to the best membranes, because PWP was increased without sacrificing retention. This may indicate that addressing the subtle balance between hydrophilic and hydrophobic contributions via further changes to the fabrication conditions, e.g., the coagulation bath composition, may lead to UF membranes breaking the trade-off between permeance and selectivity.

For PPSU, almost all additives showed an increase in PWP at retention greater than 70%, but the dextran retention of the reference membrane was 96.5% (Figure 9). The easier destabilization of the PPSU solution seems to lead to an increase in PWP already at small fractions of hydrophilic component. Rheology data revealed the strongest effects on PPSU solution destabilization by P2.5L (Figure 5). However, it is also evident that all membranes with higher PWP had lower dextran retention. Considering this trade-off without exception implies that the modification of the PPSU membrane should lead to other improvements, notably in terms of anti-fouling properties, in order to make that approach potentially viable for applications.

### 3.4. Adsorptive NOM Fouling

Flower soil extract was used to determine the fouling behavior of the membranes. This foulant represents the NOM composition of surface water well. According to the literature [23], the largest portion of the extract consists of various humic acids (71.8%). Degradation products of these humic acids (13.8%) and low-molecular-weight neutral substances (12.3%) are other bigger fractions. The remaining 1.2% is formed by low-molecular-weight acids (0.2%) and other biopolymers (1%). This is a very challenging system with which to assess the fouling propensity of ultrafiltration membranes that is only used by very few researchers [21,23,50]. A quantitative comparison with other literature data is not possible, but the system is very valuable to assess differences between different membranes that are evaluated in parallel.

**Figure 10:**
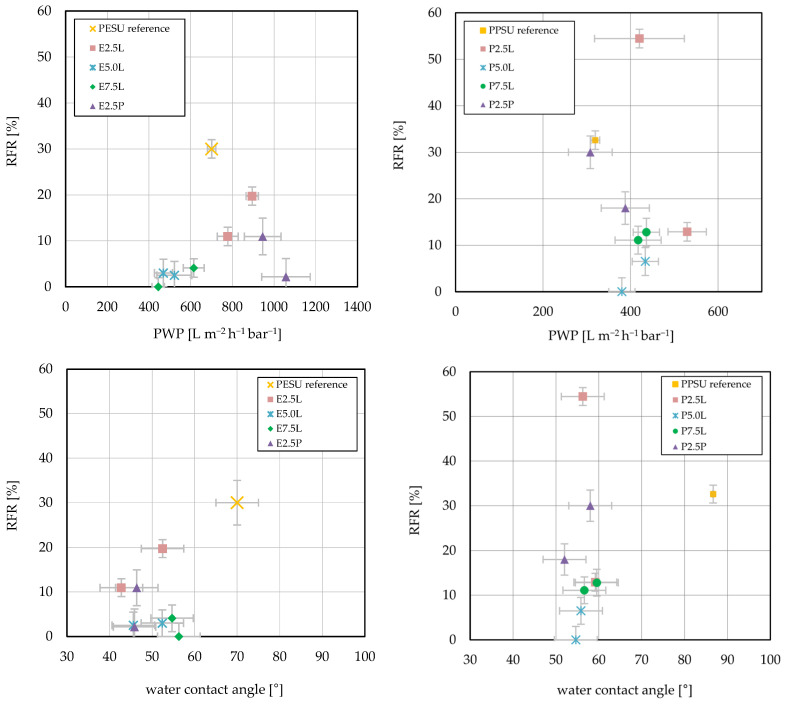
(**Top**): RFR versus PWP for PESU (left) and PPSU (right); (**bottom**): RFR versus water contact angle for PESU (left) and PPSU (right).

Anti-fouling properties, with their impact on reduction in permeability, depend on surface hydrophilicity, charge, topography and pore structure. As described in Section 3.2, no independent quantitative SEM-based analysis of surface pore structure (pore size and porosity) that would enable a comparison of the different membranes could be established. Hence, the key indicators for differences in pore structure are derived from evaluation of ultrafiltration performance. Results are shown in Figure 10, where RFR values are plotted versus either PWP or water contact angle. By using an appropriate dilution of the extract, an RFR value of 30% is obtained for the PESU reference membrane. For all membranes prepared with additive, the RFR values are significantly lower, but differences as functions of additive structure are also observed. For the Lutensol-based additives that led to higher PWP, higher RFR was also observed. With longer PES chains in the additive, PWP and RFR were lower. For the Pluronic-based additive at high concentration, higher PWP with very low RWR was found.

For the PPSU reference, an RFR of 32% was found. The slightly higher value can be related to the significantly higher contact angle, i.e., higher hydrophobicity, compared with the PESU membrane (cf. Section 3.2). In contrast to the PESU/Lutensol-based membranes, not all additives showed an improvement in fouling behavior. Again, additives with longer PPSU blocks showed a better anti-fouling effect than the 2.5 k PPSU-Lutensol variants. However, no clear dependency of the RFR on the PWP was found. For the 5.0 k PPSU variation, an RFR below 10% was found in both proportions, and RFR became minimal (<5%) for the membrane with lower additive content in the casting solution. For P7.5L, both RFR data were similar, i.e., 11–13%. Membranes with Pluronic additives did not show significant improvements in anti-fouling behavior, especially compared to the PESU-based analogous membranes.

Overall, neither for PESU nor for PPSU, a clear correlation of RFR and water contact angle was found. Hence, more specific cases should be discussed. For PESU, a reduction in fouling was achieved in all membranes. For Lutensol, lower permeability with simultaneous low fouling was found. For PPSU, not all membranes showed a reduction in RFR. However, unlike for PESU, Lutensol-based additives with longer PPSU segments led to less fouling while increasing permeability. Less pronounced fouling reduction was observed for P2.5P compared with E2.5P. Therefore, fouling reduction for membranes with decreased permeability is probably due to the smaller pore size. Accordingly, for membranes with increased permeability, other factors must be considered.

For PESU, all modified membranes showed a reduction in the water contact angle, indicating a more hydrophilic surface (cf. Figure 8). At the same time, the RFR was decreased for all membranes. Besides the reduced permeability, hydrophilicity seems to be another influence for Lutensol-based additives with longer PESU blocks, as they showed the lowest fouling tendency. However, no clear correlations between fouling reduction and permeability or water contact angle were observed. Direct spectroscopic detection of the additives at the membrane surface is associated with difficulties, due to the low concentration of the additives and the additional influence of post-treatment. However, with increased hydrophilicity in combination with a simultaneous increase in permeability, an effect of the additives at the surface can be assumed.

For PPSU, the permeability increased for Lutensol in combination with 2.5 k PPSU with a simultaneous increase in hydrophilicity. However, the performance was not improved in terms of mitigating adsorptive fouling. Apparently, the influence of the additives on the surface is not strong enough to compensate for the increase in pore size. Since PPSU is an overall more hydrophobic material and the casting solutions are destabilized by additive addition, faster precipitation and thus possible defect sites can be assumed. These may be responsible for the increase in fouling tendency. With larger PPSU block lengths, the reduction in adsorptive fouling is successful.

## 4. Conclusions

This study provides a comprehensive comparison between the alternative membrane polymer PPSU and the established material PESU, in combination with different PPSU- or PESU-based PEO-containing amphiphilic block copolymers, with respect to membrane formation and the effects of additives on membrane structure, ultrafiltration performance and NOM fouling. At the same polymer fraction, PPSU solutions have higher viscosity than PESU solutions. The amphiphilic additives destabilize the casting solution; this effect is more dominant in PPSU systems. Membrane formation from analogous casting solutions yields higher permeance for PESU-based membranes compared with PESU-based membranes. In PESU systems, additives with longer blocks lead to reduction in permeance at maintained dextran retention. For PPSU, interesting additive candidates leading to overall improved ultrafiltration performance, i.e., higher permeance at the same dextran retention, can be identified. PPSU-based membranes have higher water contact angle than PESU-based ones. The PEO-containing additives reduce the water contact angle in all cases. With flower soil extract as a model for NOM in surface water, successful reduction in adsorptive membrane fouling could be demonstrated. Lutensol-based additives are especially promising. By screening the library of amphiphilic block copolymers in a strategic approach, a customized membrane modification portfolio is established. Block copolymer additives with shorter PESU or PPSU blocks are more likely to act as pore-formers, additives with longer PESU or PPSU blocks seem to accumulate on the membrane surface, promoting hydrophilization and anti-fouling function of PEO-containing blocks. However, detection of additives on the membrane surface is difficult due to the oxidative post-treatment as part of membrane fabrication. With this study, PPSU is directly compared to the industrially established PESU. As known from the literature and confirmed in this work, the lower water permeability of PPSU-based membranes poses challenges regarding ultrafiltration performance. By addition of low amounts of an amphiphilic block copolymer, a PPSU membrane with PESU filtration performance can be obtained, making it an interesting candidate for water filtration applications, as the lifetime of the membrane module is expected to be longer compared with PESU. Parallel work has demonstrated that hollow-fiber membranes can be fabricated from these polymer systems [21] and that interesting candidates in terms of combinations between base polymer and functional additive that have been identified in the laboratory scale flat-sheet format yield promising performance when evaluated as hollow-fiber membranes in long-term filtration experiments including cleaning cycles [23]. Overall, PPSU in combination with PPSU- and PEO-containing block copolymer has significant potential as an alternative membrane polymer in comparison to the established PESU.

## Figures and Tables

**Table 1:** Overview of the additives used in this study with their PEO fraction according to ^1^H NMR spectroscopy.

PEO-Containing Building Block	PESU *	PEO Fraction According to NMR [wt.%]	PPSU *	PEO Fraction According to NMR [wt.%]
Lutensol	E2.5L	56.7	P2.5L	52.4
Lutensol	E5.0L	54.9	P5.0L	36.9
Lutensol	E7.5L	38.1	P7.5L	29.1
F127	E2.5P	64.9	P2.5P	54.8

* Abbreviations for the block copolymer: “E” or “P” stands for PESU or PPSU; the numbers represent the average molecular weight of the PESU or PPSU block in kg mol^−1^, and “L” or “P” stands for Lutensol or Pluronic P127.

**Table 2:** Overview of dope solution composition.

	Fraction [wt.%]		
	Reference	Low Additive Content	High Additive Content
Membrane base polymer (PESU/PPSU)	19	18.4	17.4
Additive (block copolymer)	0	0.4	1.6
PVP	6	6	6
Non-solvent ^1^	10	10	10
Solvent (NMP)	65	65	65

^1^ Non-solvent: For PESU-based dope solutions glycerol is used, and for PPSU-based dope solutions, 1,2-propanediol is used.

**Table 3:** Hansen solubility parameters for polymers, solvents and non-solvents [27,28,29,30,31].

Material	$\delta_{d}$	$\delta_{P}$	$\delta_{h}$	δ	Δδ PESU	Δδ PPSU
PPSU	18.7	17.5	7.9	26.8	-	
PESU	19.6	10.8	9.2	24.2	-	
PVP	16.1	12.1	8.7	21.2	3	5.6
NMP	18	12.3	7.2	22.9	1.3	3.9
Water	15.5	16.0	42.3	47.8	−23.6	−21
Glycerol	17.4	12.4	29.3	36.3	−12.1	−9.5
1,2-Propanediol	16.8	9.4	23.3	30.2	−6	−3.4
Poly(ethylene oxide)	17.0	10.7	8.9	22.0	2.2	4.8

## Data Availability

Data are available from the authors upon reasonable request.

## Supplementary Materials

The following supporting information can be downloaded at: https://www.mdpi.com/article/10.3390/membranes16050178/s1, Figure S1: Exemplary series of samples from cloud point titration; Figure S2: Arrhenius plot for viscosity at 1 s-1 in the range of 20 °C to 60 °C, to analyze inter- and intramolecular interactions for PES and PPSU; Figure S3: SEM images of the top surfaces of PESU and PPSU membranes; Figure S4: Zetapotential measurements for PES and PPSU reference membrane.

## Abbreviations

The following abbreviations are used in this manuscript:

DI	Deionized
HSP	Hansen Solubility Parameter
Mw	Average Molecular Weight
NIPS	Non-Solvent-Induced Phase Separation
NOM	Natural Organic Matter
PEO	Poly(ethylene oxide)
PESU	Poly(ether sulfone)
PPO	Poly(propylene oxide)
PPSU	Poly(phenylene sulfone)
PVP	Polyvinylpyrrolidone
PWP	Pure Water Permeance
R	Retention
RFR	Relative Flux Reduction
RT	Room Temperature
SEM	Scanning Electron Microscopy
TC	Total Carbon Content
TIC	Total Inorganically Bound Carbon
TOC	Total Organic Carbon
UF	Ultrafiltration
