# Supplementary material for: Comparison of Phase Separation and Membrane Formation Behavior of Novel Amphiphilic Block Copolymers for Anti-Fouling Improvement of Ultrafiltration Membranes
Source: Membranes (Basel). 2026 May 19;16(5):178. doi: 10.3390/membranes16050178 (PMC13208665; doi:10.3390/membranes16050178)
Supplement: Supplementary file 1 [file membranes-16-00178-s001.zip › membranes-4322550-supplementary.pdf]

Supplementary Information for

Comparison of phase separation and membrane formation behavior for novel amphiphilic block copolymers for anti-fouling improvement of ultrafiltration membranes

Inga Frost [1], Oliver Gronwald [2], Mathias Ulbricht [1*]

[1] Universität Duisburg-Essen, Lehrstuhl für Technische Chemie II, Universitätsstr. 7, 45141 Essen, Germany
[2] BASF SE, Performance Materials, Research Ultrason, G-PM/PU, 67056 Ludwigshafen am Rhein, Germany
[*] Correspondence: mathias.ulbricht@uni-essen.de

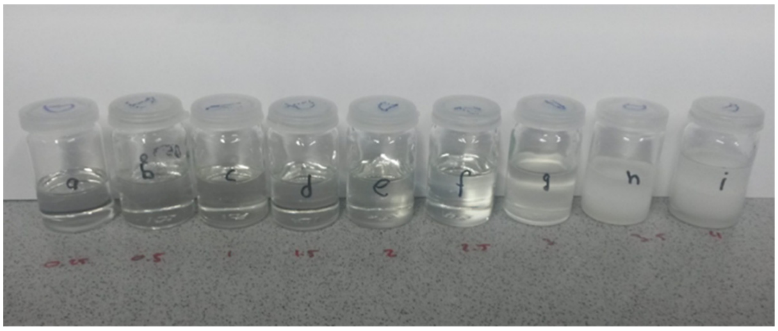

Figure S1. Exemplary series of samples from cloud point titration.

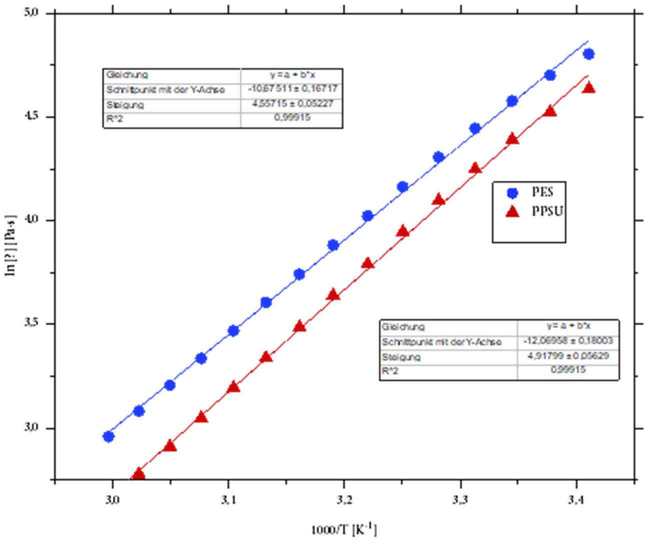

Figure S2. Arrhenius plot for viscosity at 1 s^{-1} in the range of 20 °C to 60°C, to analyze inter- and intramolecular interactions for PES and PPSU.

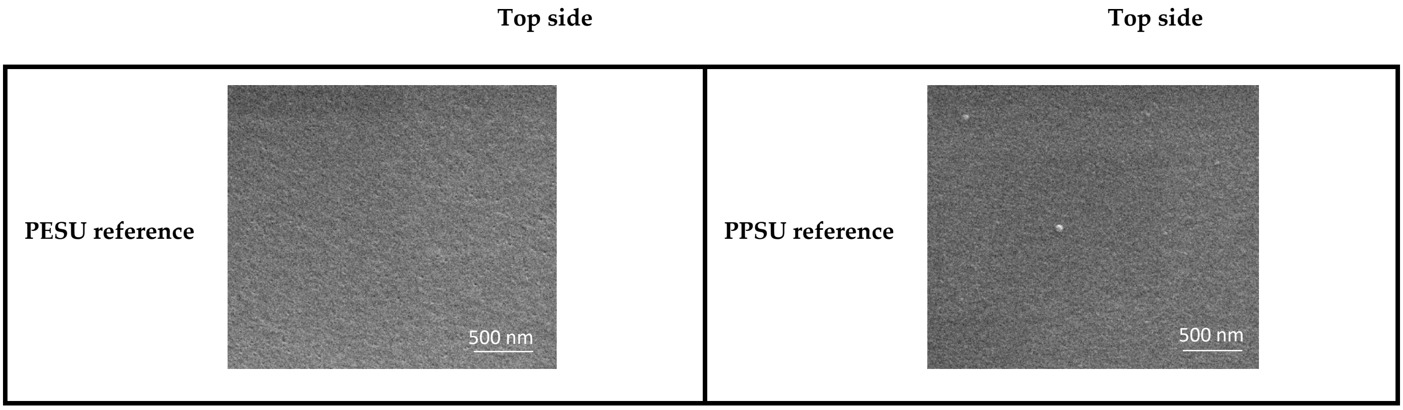

Figure S3. SEM images of the top surfaces of PESU and PPSU membranes.

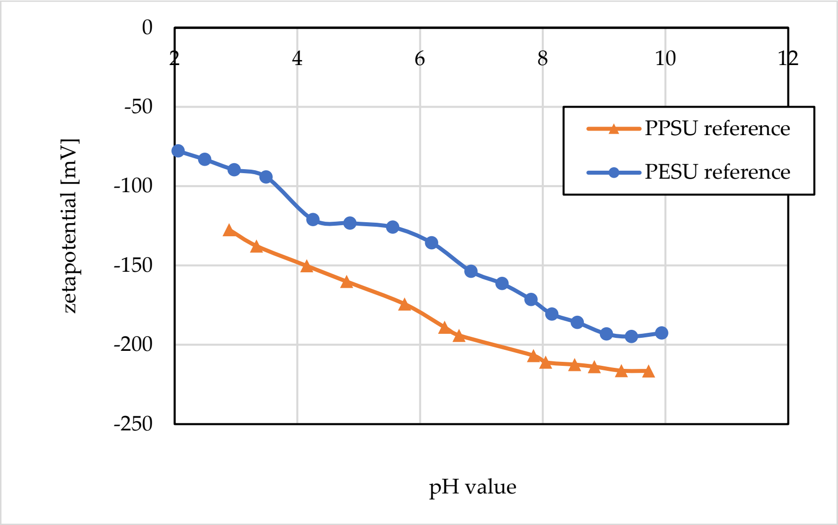

Figure S4. Zetapotential measurements for PES and PPSU reference membrane.
